# The human jejunum has an endogenous microbiota that differs from those in the oral cavity and colon

**DOI:** 10.1186/s12866-017-1059-6

**Published:** 2017-07-17

**Authors:** Olof H. Sundin, Antonio Mendoza-Ladd, Mingtao Zeng, Diana Diaz-Arévalo, Elisa Morales, B. Matthew Fagan, Javier Ordoñez, Philip Velez, Nishaal Antony, Richard W. McCallum

**Affiliations:** 1 Department of Biomedical Sciences, Texas Tech University Health Sciences Center at El Paso, El Paso, 79905 TX USA; 2 Department of Internal Medicine, Division of Gastroenterology, Texas Tech University Health Sciences Center at El Paso, El Paso, 79905 TX USA; 3 Biostatistics Unit, Texas Tech University Health Sciences Center at El Paso, El Paso, 79905 TX USA

**Keywords:** Metagenomics, Microbiome, Small intestine, Jejunum, Bacterial load

## Abstract

**Background:**

The upper half of the human small intestine, known as the jejunum, is the primary site for absorption of nutrient-derived carbohydrates, amino acids, small peptides, and vitamins. In contrast to the colon, which contains 10^11^–10^12^ colony forming units of bacteria per ml (CFU/ml), the normal jejunum generally ranges from 10^3^ to 10^5^ CFU per ml. Because invasive procedures are required to access the jejunum, much less is known about its bacterial microbiota. Bacteria inhabiting the jejunal lumen have been investigated by classical culture techniques, but not by culture-independent metagenomics.

**Results:**

The lumen of the upper jejunum was sampled during enteroscopy of 20 research subjects. Culture on aerobic and anaerobic media gave live bacterial counts ranging from 5.8 × 10^3^ CFU/ml to 8.0 × 10^6^ CFU/ml. DNA from the same samples was analyzed by 16S rRNA gene-specific quantitative PCR, yielding values from 1.5 × 10^5^ to 3.1 × 10^7^ bacterial genomes per ml. When calculated for each sample, estimated bacterial viability ranged from effectively 100% to a low of 0.3%. 16S rRNA metagenomic analysis of uncultured bacteria by Illumina MiSeq sequencing gave detailed microbial composition by phylum, genus and species. The genera *Streptococcus, Prevotella, Veillonella* and *Fusobacterium,* were especially abundant, as well as non-oral genera including *Escherichia, Klebsiella*, and *Citrobacter*. The jejunum was devoid of the genera *Alistipes, Ruminococcus*, *Faecalibacterium*, and other extreme anaerobes abundant in the colon. In patients with higher bacterial loads, there was no significant change in microbial species composition.

**Conclusions:**

The jejunal lumen contains a distinctive bacterial population consisting primarily of facultative anaerobes and oxygen-tolerant obligate anaerobes similar to those found in the oral cavity. However, the frequent abundance of Enterobacteriaceae represents a major difference from oral microbiota. Although a few genera are shared with the colon, we found no evidence for retrograde movement of the most abundant colonic microbes to the jejunum. Some individuals had much higher bacterial loads, but this was not correlated with decreases in bacterial species diversity or other evidence of dysbiosis.

**Electronic supplementary material:**

The online version of this article (doi:10.1186/s12866-017-1059-6) contains supplementary material, which is available to authorized users.

## Background

**Fig. 1:**
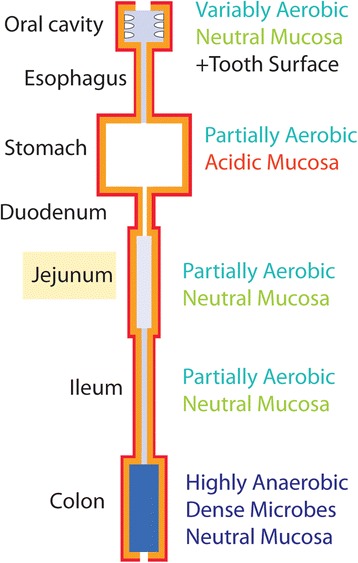
Environments of the alimentary tract. Diagram of the human alimentary tract showing the jejunum and its environment in relation to other regions. *Red outline* indicates vascularized gut lining. *Darker blue* indicates higher density of luminal bacteria. *Numbers* indicate reported bacterial densities for different regions

The jejunum is a structurally and functionally distinct region of the alimentary tract that spans the upper half of the small intestine (Fig. 1). Its inner surface contains an extensive array of vascularized villi that efficiently absorb carbohydrates, amino acids, small peptides, and vitamins [1]. In contrast to the colon, which has a dense population of 10^11^–10^12^ bacteria per ml [2], the jejunal lumen of healthy individuals has a relatively sparse bacterial population of 10^3^–10^5^ CFU/ml [3–5]. The composition and source of this jejunal microbiota remains an open question. One possibility is that its bacteria are continuously replenished by bacteria passing from higher levels of the alimentary tract, such as the oral cavity. However, few bacteria normally survive this journey. Acid and enzymes secreted by the stomach have a strong bacteriocidal effect on salivary or food-associated microbes. Fewer than 10^1^–10^2^ CFU/ml typically pass down the alimentary tract to the duodenum and jejunum [1, 2]. Another possible source of microbes is the wall of the jejunum itself, where bacteria are tightly associated with the villi of the intestinal mucosa [6]. The glycoprotein matrix on the surface of these villi could play a role similar to glycoprotein-rich mucosal layers of the colon, which foster a renewable seed population of bacteria in the lower gut [7]. Yet another possible source is the colonic lumen, which normally contains a total of 10^12^–10^13^ bacteria [2]. Continuous downward peristalsis and restriction of retrograde flow by the iliocaecal valve play an active role in keeping the upper jejunum free of colonic bacteria. However, given the enormous population of bacteria in the colon, even a very small retrograde migration into the small intestine would be sufficient to greatly increase the bacterial population of the jejunum. During acute and chronic infection of several levels of the gut, there is a complex interaction between pathogen and the resident bacteria. Better understanding of the composition and dynamics of this resident microbial population will be crucial in developing methods to restore it to a healthy state via dietary changes or microbial transplantation with defined populations of bacteria [8, 9].

Given its location deep within the human body, retrieving bacteria from the jejunum is technically much more difficult than sampling oral or fecal microbes. As a result, there have been very few studies of the human jejunal bacteria [3, 4]. Microbial sampling of the intestinal lumen has usually been achieved by passing a tube or other collection device to the jejunum via the oral cavity, esophagus, stomach and duodenum [10–12]. In these earlier studies, X-ray fluoroscopy was often used to determine position of the probe within the gut. With endoscopes, entry of the probe into the jejunum can be monitored by fiber optic imaging of anatomical landmarks. In preparation for such endoscopic sampling, the mouth and teeth need to be thoroughly cleaned and the endoscopy tube closed in order to avoid possible contamination by oral bacteria. Live bacterial cultures retrieved from the human proximal jejunum have included the gram-positive genera *Streptococcus* and *Veillonella,* as well as the gram-negative enterobacterial genus *Escherichia* and *Enterobacter* [10–12]. In recent years, culture-independent microbial identification via high-throughput DNA sequencing of 16S ribosomal RNA genes has transformed our ability to investigate the complex bacterial populations of the human body [13]. Because this new methodology provides efficient and reliable detection of many bacteria, including those that are not readily grown in culture, it has been proposed that a complete survey of jejunal bacteria will require such a culture-independent approach [3, 12].

Currently, there are only two published 16S rRNA metagenomic studies of bacteria from the human jejunum [14, 15], but neither of these sampled the jejunal lumen. In each case, a tissue biopsy was excised from the wall of the small intestine and then washed extensively to select only those bacteria that were tightly adherent to the mucosal surface. The first of these studies utilized low-throughput Sanger DNA sequencing of plasmid-cloned 1.5 kb 16S rRNA gene amplimers to identify bacteria from intestinal tissue biopsies of a single individual [14]. This study identified *Streptococcus mitis* as the most abundant bacterial species in the jejunum. The second study [15] employed Roche 454 Next Generation DNA deep sequencing of 16S rRNA amplimer libraries to identify bacteria in jejunal tissue biopsies of a cohort of patients with irritable bowel syndrome (IBS). Although the bacterial assortment varied between individuals, the mucosal microbiota in normal, asymptomatic controls was statistically indistinguishable from that of patients with IBS. One interpretation of these results is that mechanisms governing bacterial species composition of the jejunum are surprisingly robust, since species composition appeared unaffected by the severe inflammatory pathology of IBS [15].

In the current exploratory study, we have built upon this earlier work with some new approaches. Our study is the first to carry out high-throughput 16S metagenomic analysis of bacteria retrieved directly from the jejunal lumen. It is also the first study to simultaneously quantify live colony forming units and bacterial DNA in the human jejunum. Earlier studies that used bacterial culture to quantify bacterial load within the jejunal lumen were generally motivated by clinical interest in a gastrointestinal dysfunction that is often associated with an overgrowth of bacteria in the small intestine [3]. For this reason, we considered it important to measure the density of bacteria in the lumen, as well as to determine its composition profile by phylum, genus and species. In addition, while many metagenomic studies focus on phylum and genus level classification [13], we have attempted, where possible, to characterize jejunal bacteria at the species level.

## Methods

### Research subjects

We enrolled 5 male and 15 female subjects, aged 26 to 79, with a median age of 53. Research subject recruitment and written informed consent were performed under authorization of the Texas Tech University Health Sciences Center at El Paso Institutional Review Board according to principles of the Declaration of Helsinki. The subjects were under clinical evaluation for moderate gastrointestinal symptoms, including bloating, gas, or irregular bowel habits [4]. Given the invasive nature of enteroscopy, our IRB committee required this medical justification for the procedure. Criteria for exclusion from the study were: any evidence of acute gastrointestinal infection, antibiotic use within 4 weeks, inflammatory bowel disease, malignancy of any kind, small bowel obstruction, small bowel fistulas, bariatric surgery, Billroth procedures Types 1 and 2, ileostomy, as well as small bowel or colonic resections.

### Collection of jejunal microbes

Samples from the jejunal lumen were collected after an overnight fast using an enteroscopic wash procedure similar to an earlier study [16]. Just before the procedure, subjects brushed their teeth extensively and used mouthwash to avoid contamination of the enteroscopy probe during its passage through the mouth. Samples were collected using an Olympus SIF-Q180 Enteroscope, which was capped to close the air column during passage. Once the probe reached 20 cm past the ligament of Treitz into the proximal jejunum, 50 ml of sterile, deionized water was injected into the lumen. Lavage of the local lumen continued for 1 min, after which 40 ml of fluid was retrieved by aspiration through the instrument and stored sterile on ice. Centrifugation-clarified jejunal aspirates were measured with a Fisher Traceable® precision electrical conductivity meter. Conductivity was compared with that of a reference solution of 142 mM NaCl, 4.5 mM KCl and 0.5 mM CaCl_2_ . This reference solution was modeled on electrolytes within the human jejunum, which show minimal subject-to-subject variation in fasting individuals [17]. Conductivity decreases relative to this reference solution allowed us to estimate the lavage dilution factor, and calculate the original microbial density in undiluted luminal fluid.

### Anaerobic and aerobic microbial culture

Bacteria were plated within 3 h of collection in 10-fold dilution steps, following standard procedures [18]. Anaerobic cultures were plated on CDC blood agar, KV blood agar (kanamycin-vancomycin), or phenylethyl alcohol blood agar (Thermo Scientific, Lenexa, KS). Plates were incubated at 37 °C in anaerobic Gas Pak 100 System Jars (Becton Dickinson, Franklin Lakes, New Jersey) and counted after 48 h. Aerobic cultures were plated on trypticase soy agar with 5% sheep blood or on Levine eosin-methylene blue agar (Becton Dickinson), and colonies counted after 24 h incubation at 37 °C. Standard summation of bacterial colony counts from all 5 culture conditions was followed by normalization to undiluted luminal fluid. This provided total bacterial CFU per ml. To spot-check the identity of cultured bacteria, DNA from 45 random colonies, 4 per patient of the first 12 patients, was PCR amplified with 16S rRNA gene V1-V3 PCR primers [19], using the same primers and PCR conditions as QPCR (see below). Amplimers were Sanger sequenced on both strands, and NCBI Blast searches of assembled amplimer sequences used to identify the bacterial genus and species.

### DNA extraction and bacteria-specific QPCR analysis

Microbes and cellular debris of the jejunal aspirate were pelleted by centrifugation for 10 min at 10,000 x g and stored at −80 °C. The Power Soil Microbial DNA Isolation Kit (MoBio Laboratories, Carlsbad, CA) was used to mechanically disrupt bacteria and purify DNA from the pellet. A sample of saliva containing oral bacteria was processed as a positive control. Yield of bacterial DNA was estimated by quantitative PCR (QPCR) carried out in triplicate using an Applied Biosystems Step One Plus thermal cycler with SYBR Green detection. QPCR amplification of the V1-V3 hypervariable regions of the 16S rRNA gene with the degenerate pan-bacterial primers 27ForM-20mer: AGAGTTTGATC(A,C)TGGCTCAG and 533RevK-19mer: TTACCGCGGC(G,T)GCTGGCAC [19] was performed at a stringent annealing temperature of 59 °C. Crossing threshold (C_t_) values were converted to microbial DNA concentration and *E. coli* genome equivalents with reference to a standard dilution series of *E. coli* K12 DNA. This number was multiplied by 1.75 to adjust for the fact that an *E. coli* genome has 7 identical 16 s rRNA genes, while 4 per genome are found in *Streptococci* and most other gut microbes.

### 16 s rRNA gene sequencing and metagenomic analysis

Illumina 16 s rRNA gene libraries were generated for each patient sample by 15 or more cycles of PCR amplification with 16S Illumina Amplicon primers at an annealing temperature of 55 °C. Cycle number was adjusted in accordance with the abundance of bacterial DNA in the sample [20]. Illumina Amplicon primer sequences were:

- Amp_for: tcgtcggcagcgtcagatgtgtataagagacaGCCTACGGG (A,C,G,T) GGC (A,T)GCAG
- Amp_rev: gtctcgtgggctcggagatgtgtataagagacagGACTAC(A,C,T) (A,C,G) GGGTATCTAATCC

The 3′-terminal nucleotides (upper case) represent degenerate DNA sequences that match extremely conserved sequences flanking the V3 and V4 hypervariable regions of all eubacterial 16S rRNA genes [20], while the 5′ adaptor sequences (lower case) allow re-amplification of each library with a unique combination of bar-coded Illumina Nextera forward and reverse adaptor-primers. Each library was marked by a unique pair of bar-coded primers, and 11 libraries were pooled into a single DNA template preparation for the Illumina MiSeq [20]. For each patient, Illumina MiSeq sequencing (SeqMatic LLC, Fremont CA) yielded roughly 300 bp of sequence from forward and reverse strands of each molecular cluster template to assemble a ~460 bp 16S V3-V4 region DNA sequence. Using bar-coded sequence tags, data was sorted back into individual patient samples, and analyzed with 16S rRNA metagenomic work flow features of the updated MiSeq Reporter v2.5 software package [21]. Each patient sample yielded between 3 × 10^4^ to 1.3 × 10^6^ high quality bacterial template reads. Custom Perl scripts retrieved raw DNA sequence data from annotated individual sequence reads. In some cases 10 or more examples of a sequence were assembled with Clustal W to obtain a consensus. Sequences of individual reads or consensus sequences of multiple reads were used to manually query NCBI genomic databases to confirm genus and species assignments made automatically by the Illumina Reporter v2.5 software. Open-source R Consortium software (www.r-project.org) was used for principal component analysis and other statistical measures.

### Statistical analysis

All basic statistical analyses were performed using R version 3.2.5 software (released 2016), obtained from the open-source R Consortium (www.r-project.org) and running on Mac OS X version 10.10. The Pearson linear correlation coefficient was calculated for 2-variable data where one or both axes were log-transformed. *P*-values were determined for the null hypothesis of no correlation between the variables. For 5-variable phylum composition data, principal components analysis was carried out with pca, princomp, plot and biplot functions of the R statistical package.

## Results

### Microbial culture

Total bacterial colony counts in the jejunal lumen ranged widely, from 5.8 × 10^3^ to 8.0 × 10^6^ CFU/ml (Table 1, column 7). The culture data from individual subjects showed considerable heterogeneity in the ratio of bacterial colonies appearing in 5 different aerobic and anaerobic culture conditions, suggesting an underlying heterogeneity in species composition. The data in Table 1 shows that rich aerobic medium, Tryptose-Blood agar (column 5) yielded many colonies for all patients. In contrast, the coliform-selective Levine-EMB agar plates (column 6) grew many colonies for fewer patients. Anaerobic CDC-Blood agar (column 7), which supports a wide range of facultative and obligate anaerobes, generally grew the largest numbers of colonies. Similar numbers of colonies usually grew on PEA-Blood (column 8), a medium selective for both gram-positive and gram-negative obligate anaerobes. Very few colonies grew on KV-Blood (column 9), a medium selective for gram-negative obligate anaerobes. Overall, this suggested that most bacteria retrieved from the jejunum preferred to grow in an anaerobic environment, and that these anaerobes were primarily gram-positive.

**Table 1:** Microbial Culture and QPCR Analysis of Jejunal Lumen

Patient	Bact. DNA	Colonies	Viability	Aerobic Culture CFU		Anaerobic Culture CFU		
	BGE/ml	CFU/ml	CFU/BGE	Trypt-Blood	Levine-EMB	CDC-Blood	PEA-Blood	KV-Blood
				%	%	%	%	%
A	3.1 × 10^7^	1.6 × 10^5^	0.005	7.9	0.0	52.6	39.5	0.0
B	2.9 × 10^7^	4.7 × 10^5^	0.016	6.8	69.1	3.20	14.3	6.6
C	2.4 × 10^7^	4.2 × 10^5^	0.018	18.1	27.2	34.4	20.0	0.3
D	1.3 × 10^7^	7.9 × 10^5^	0.061	49.7	9.9	34.8	5.0	0.6
E	1.3 × 10^7^	3.1 × 10^5^	0.024	26.3	0.8	41.3	30.1	1.5
F	8.5 × 10^6^	2.2 × 10^4^	0.003	2.7	0.0	43.3	54.0	0.0
G	7.9 × 10^6^	1.1 × 10^5^	0.014	29.7	0.0	48.8	21.6	0.0
H	5.5 × 10^6^	5.2 × 10^6^	0.945	9.7	1.0	71.1	18.1	0.1
I	5.2 × 10^6^	3.1 × 10^4^	0.006	21.3	0.0	65.6	13.1	0.0
J	4.7 × 10^6^	1.5 × 10^6^	0.319	5.1	0.1	43.6	51.2	0.0
K	4.5 × 10^6^	3.0 × 10^6^	0.667	27.1	25.1	36.8	11.0	0.0
L	3.2 × 10^6^	7.9 × 10^6^	2.469	34.5	0.6	23.7	39.8	1.4
M	3.2 × 10^6^	2.2 × 10^4^	0.007	6.2	0.0	29.2	64.6	0.0
N	2.3 × 10^6^	2.0 × 10^5^	0.087	5.7	0.0	85.7	8.6	0.0
O	2.1 × 10^6^	8.4 × 10^5^	0.400	16.2	0.0	68.1	12.7	3.0
P	1.3 × 10^6^	2.8 × 10^4^	0.022	11.1	0.0	74.1	14.8	0.0
Q	1.0 × 10^6^	6.6 × 10^4^	0.066	6.4	5.9	14.0	73.7	0.0
R	7.3 × 10^5^	5.7 × 10^3^	0.008	21.4	0.0	71.4	7.1	0.1
S	2.8 × 10^5^	9.9 × 10^3^	0.035	14.2	0.0	62.7	18.9	4.2
T	1.5 × 10^5^	4.2 × 10^4^	0.280	7.6	0.1	75.9	15.1	1.3

Bacterial yields have been normalized to bacteria per ml of standard undiluted jejunal contents. Results were calibrated with a standard dilution series of purified *E. coli* DNA. Column 1 is the patient designation, ranked according to total live bacteria. Column 2 is the bacterial genome number estimated by quantitative PCR analysis of extracted DNA using pan-bacterial PCR primers for 16S rRNA genes. Column 3 represents live bacterial colonies, consisting of the sum of CFU/ml for all 5 culture media. Column 4 represents the viability fraction of a given sample. This is the ratio of CFU/BGE. Sample L is likely greater than 1 because of errors in the two measurements, or assumptions regarding average 16S gene number of the bacteria. Columns 5 through 9 represent the percent of total colony forming units (CFUs) counted on different culture media

### Comparison of live bacterial counts with bacterial DNA

In preparation for the identification of bacteria by DNA sequencing, we measured their total levels by quantitative PCR of bacterial 16S rRNA genes. The pan-bacterial oligonucleotide primers used for QPCR were highly selective for eubacterial 16S rRNA, and did not amplify mammalian, plant, fungal or archaeal rRNA genes [19]. QPCR provided a direct, culture-independent measure of the number of genomes of bacterial DNA. Comparison of QPCR measurements with OD_260_ total DNA yield indicated that in most cases, less than 1% of total purified DNA was of bacterial origin (not shown). To examine this non-bacterial DNA, a set of 16 “Unclassified” sequence reads were retrieved from the raw data set of the Illumina high throughput DNA sequences, and examined by NCBI-BLAST homology search. Each of these reads proved to be close matches to random, non-ribosomal portions of the human genome, apparently amplified because of chance annealing to the Illumina 16S rRNA-homologous primers used for Illumina MiSeq library construction. This human DNA probably comes from epithelial cells that are normally shed from the gut lining.

**Fig. 2:**
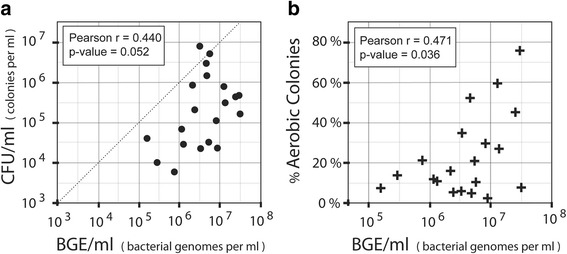
Viable colony counts and fraction of aerobic colonies. **a** Viability. Bacterial density (BGE/ml: bacterial genome equivalents per ml) derived from pan-bacterial PCR of DNA (x-axis) are compared with viable colony counts (CFU/ml). Results are plotted on log-log scales, with each point representing a single research subject. The *dotted line* represents theoretical 100% colony-forming viability of bacteria detected by DNA. **b** Aerobic Fraction. Bacterial density in BGE units/ml (x-axis) is plotted on a log scale against the fraction of aerobic colonies, as derived from Table 1. In each case the Pearson correlation coefficient r and *p*-value for the null hypothesis of *r* = 0 were calculated

The jejunal lumen of our subjects contained from 1.5 × 10^5^ to 3.1 × 10^7^ bacterial genome equivalents per milliliter (BGE/ml). These DNA-based bacterial counts were nearly always larger than the live colony counts, and distributed over a ~ 7-fold narrower range (Table 1). When we compared QPCR results with viable counts for each sample, we found large differences in apparent viability of the bacterial genomes detected in extracted DNA. When graphed for each subject (Fig. 2a), there was considerable scatter between bacterial DNA and colony counts, with a Pearson correlation coefficient of 0.44 and a marginally significant *p*-value of 0.052. Several samples plotted close to the diagonal line, which represented one CFU per bacterial genome (Viability = 1.0). However, most samples exhibited viability below 0.1, with a low of 0.003, where roughly one out of 300 bacterial DNA genomes corresponded to a colony-forming bacterium (Table 1). For subject L, the viability was an “impossible” 2.47. In this case, most of the live bacteria may have been facultative anaerobes, which grow readily on both aerobic and anaerobic media. If we take this into account and count only the rich medium aerobic cultures, we still have a 0.85 viability fraction, which is very high. Such inflation of cell count numbers is an inherent drawback of culture-based methods designed to include bacteria with widely varying growth requirements. The observed low levels of viability for many samples could have resulted from the inactivation of bacteria caused by environmental factors such as pH, pancreatic secretions, cold treatment after collection, or other unknown factors. Species-related differences in plating efficiency in standard culture might also have contributed variation in viability, as well as physical grouping of bacteria into doublets or larger micro-colonies.

### A larger fraction of aerobic microbes was associated with higher bacterial loads

When the percentage of aerobic colonies was plotted against the log of bacterial load (Fig. 2b) we observed that patients with higher bacterial loads tended to have a higher percentage of aerobic colonies. Although there was considerable variation between individuals, this positive correlation was statistically significant, with a Pearson correlation coefficient of *r* = 0.471 and a *p*-value of 0.036. Overall, our results suggested that subjects with higher density of total jejunal bacteria had a larger fraction of viable aerobes or facultative anaerobes.

### Phylum-level profile

**Fig. 3:**
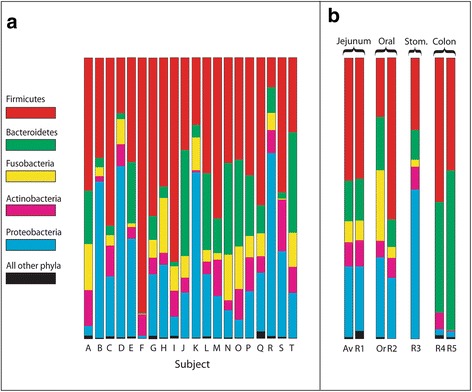
Relative bacterial abundance by phylum. **a** Metagenomic analysis. Estimates of the relative amount of microbial DNA in each patient from 5 major phyla (*colors*), and all other bacterial phyla combined (*black*). **b** Comparison of results with earlier studies. Av: Average of all patient data in Fig. 2a. Or: Oral saliva test sample. R1: Averaged jejunal phylum distribution, from reference [15]. R2: Averaged saliva phylum distribution (ref [22]). R3: Averaged phylum distribution for stomach from reference [23]. R4: Averaged adult colon phylum distribution (ref [24]). R5: Averaged colon distribution from aged individuals (ref [25])

In the metagenomic analysis, each sample generated from 5 × 10^4^ to 1.7 × 10^6^ raw DNA sequence reads, with 85% to 100% passing the Illumina quality filtering. Of these, between 37% and 99% were classified as bacterial. All samples of non-bacterial sequences that were retrieved and classified by BLAST search were found to match random regions of non-ribosomal human genomic DNA. Over 99% of confirmed bacterial 16S rRNA gene sequence reads were classified by phylum, 95% classified by genus, and 78% were further assigned to a species. Phylum distributions varied substantially between individual patients. Nearly all classified bacterial DNA sequences fell within 5 of the 28 recognized bacterial phyla. In Fig. 3a, samples have been ordered sequentially from the highest to the lowest bacterial load as determined by bacteria-specific QPCR, showing the variation in phylum composition between individuals. Figure 3b presents averaged phylum distribution data derived from our work and published studies. In general, the upper alimentary tract (oral saliva [22], stomach [23]) and jejunum [15] had significant levels of Proteobacteria and Fusobacteria, while bacteria belonging to these phyla were rare in the colon. Metagenomic analysis showed that colonic bacteria of adults were almost exclusively composed of the phyla Firmicutes and Bacteroidetes, with a small fraction of Actinobacteria and Proteobacteria [24, 25]. Composition varied substantially between individual patients, but when all 20 were averaged together (Table 2) the result was surprisingly close to the average phylum profile obtained by Dlugosz et al. in an earlier study [15] of microbes in the jejunal mucosa. The average phylum distribution was also similar to that of oral salivary bacteria [22, 26], but differed considerably from that of colonic microbiota [24, 25].

**Table 2:** Average distribution of bacterial phyla

	Jejunum	Jejunum	Oral 1	Oral 2	Colon 1	Colon 2
	Average %	Dlugosz	Guerrero-P.	Oh	Turnbaugh	Claesson
Phylum	(this report)	ref. [15]	ref. [26]	ref. [22]	ref. [24]	ref. [25]
Firmicutes	43.7	43.0	47.1	57.6	51.4	40.0
Proteobacteria	25.5	23.0	22.7	21.6	2.1	2.0
Bacteroidetes	14.4	15.0	21.2	9.8	39.5	57.0
Actinobacteria	8.1	9.3	5.0	7.1	5.9	0.4
Fusobacteria	7.8	7.0	2.9	3.9	0	0.0
all other phyla	0.5	2.7	1.1	0.0	1.1	0.6
Total	100.0	100.0	100.0	100.0	100.0	100.0

Column 1 shows the mean percent value for the 16S rRNA gene profile of 5 major phyla, and of all minor bacterial phyla combined. This is compared with similar profiles from the jejunal mucosal data of Dlugoz et al. [15], and averaged data from two studies for oral saliva (Guerrero -Preston et al. [26] and Oh et al. [22]). Colon data is from normal adults (Turnbaugh et al. [24]) and aged individuals Claesson [25]

**Fig. 4:**
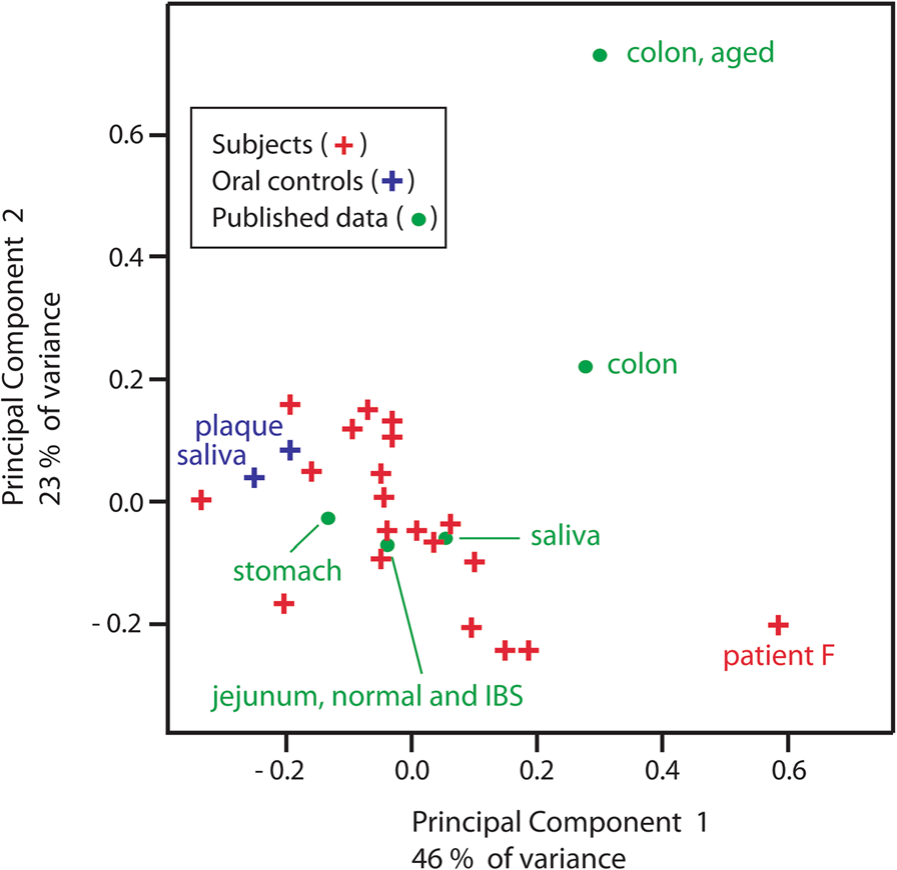
Principal components analysis of phylum composition. Phylum distribution data from individual jejunal samples (*red crosses*). Positive control oral microbes of saliva and dental plaque positive controls (*purple crosses*). *Green dots* represent averaged results from the literature: jejunum [15] saliva [22], stomach [23], adult colon [24], and geriatric colon [25], as in Fig. 3b

Figure 4 shows a principal components analysis of the phylum composition data. It illustrates that the jejunal microbiota from 19 of the 20 subjects cluster close to the oral dental plaque and saliva controls (purple crosses) as well as published data (green dots) representing averaged phylum composition of saliva [22], stomach [23], and jejunum [15]. Colonic microbiota from adults [24] and aged adults [25] have Firmicutes and Bacteroidetes-rich compositions that place them as distant outliers from the jejunal-oral cluster, which includes 19 jejunal samples. The 20th jejunal sample, from subject F, is a strong outlier from the main cluster that resembles the profile of colonic bacteria along the first principal component (x-axis), but more closely resembles oral and jejunal bacteria in the second principal component (y-axis). The “colonic” character of subject F at the phylum level is based on the fact that it contained primarily Firmicutes and had few Proteobacteria or Fusobacteria. The smaller difference in the second axis is based on the fact that its minor component was Actinobacteria rather than Bacteroidetes. Closer examination at the genus level indicated that the Firmicutes component of jejunal bacteria in subject F was not colonic in character, since it consisted primarily of the genus *Streptococcus*. *Streptococcus* is more characteristic of oral bacteria than the Clostridia, which include obligate and extreme anaerobes typically abundant in the colon.

### Proteobacteria detected by metagenomics are strongly correlated with the fraction of aerobic colonies

**Fig. 5:**
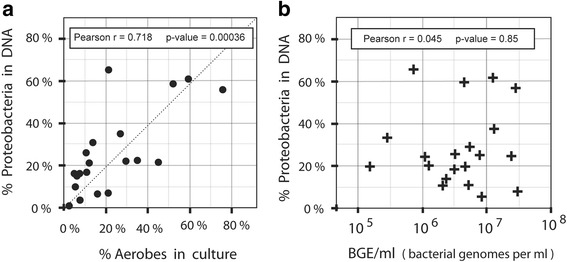
Correlation of Proteobacterial fraction with aerobic colonies. **a** The percentage of aerobic colonies observed in culture (x-axis) is plotted against the percentage of Proteobacteria among total bacterial 16S rRNA gene sequence reads (y-axis). **b** Bacterial DNA abundance, in bacterial genome units per ml, is plotted on a log scale (x-axis) against the percentage of Proteobacteria (y-axis). The Pearson correlation coefficient and *p*-value for each graph are shown in the inset

Figure 5a relates the Proteobacterial phylum composition of bacterial DNA of each subject to the percentage of aerobic colonies determined by bacterial culture (Table 1). The very strong correlation (*r* = 0.718) is highly significant at a *p*-value of 0.00036. Significant correlations were not obtained for any of the 4 other phyla, suggesting that many viable aerobes or facultative anaerobes were Proteobacteria. Surprisingly, when the fraction of Proteobacteria was compared with bacterial DNA abundance (Fig. 5b), there was no significant correlation (*r* = 0.045, *p*-value = 0.85). This suggested that the increased fraction of aerobic colonies in high bacterial load samples (Fig. 2b) was not primarily the result of increased Proteobacteria at the DNA level. The positive correlation shown in Fig. 2b may, in part, reflect better viability of aerobes at higher bacterial loads rather than microbial composition.

### Genus-level profile

Table 3 shows the average representation of the top 20 bacterial genera in the jejunal lumen of all subjects. This distribution is dominated by the facultative anaerobes *Streptococcus, Escherichia* and *Haemophilus,* which have the ability to switch to more efficient metabolism by oxidative phosphorylation in an aerobic environment. Facultative anaerobes can produce vigorous colonies on both aerobic and anaerobic media. Obligate anaerobes were also abundant in the jejunum, represented most prominently by *Prevotella, Veillonella and Fusobacterium*. Unlike extreme anaerobes, species from these genera are not poisoned by oxygen. However, they are not capable of efficient aerobic metabolism and therefore grow slowly in aerobic cultures. The Pearson correlation coefficient was calculated to determine whether there was any relationship between the fractional abundance of individual genera and the log_10_ of the bacterial load. All except 4 of the top 26 genera revealed no significant difference from the null hypothesis. The genera with individually significant differences, including *Rothia*, *Lachnoclostridium*, *Campylobacter* and *Ralstonia* all showed a negative correlation, indicating that they represented a smaller fraction of the bacterial population in samples with higher bacterial loads (Table 3). After Bonferroni correction for multiple testing, these differences do not reach a threshold of significance. Larger numbers of subjects would be necessary to resolve this conclusively. Currently, our results suggest that differences in microbial composition at the genus level are not significantly correlated with bacterial load, even though this load varies over a 200-fold range between subjects.

**Table 3:** Average composition of the jejunum by genus and its correlation with bacterial load

Rank	Composition	Pearson Correlation with Log Bacterial Load	*P*-value of Correlation	Genus	Phylum
	Jejunum				
1	28.0%	0.036	0.880	Streptococcus	Firmicutes
2	12.5%	−0.296	0.205	Prevotella	Bacteroidetes
3	6.7%	0.158	0.507	Veillonella	Firmicutes
4	6.5%	−0.236	0.316	Escherichia	Proteobacteria
5	5.7%	0.188	0.428	Fusobacterium	Fusobacteria
6	5.2%	0.163	0.492	Haemophilus	Proteobacteria
7	2.8%	0.201	0.396	Actinomyces	Actinobacteria
8	2.3%	−0.554	0.011	Rothia	Actinobacteria
9	2.3%	−0.428	0.060	Leptotrichia	Fusobacteria
10	2.0%	0.101	0.672	Gemella	Firmicutes
11	2.0%	−0.264	0.261	Neisseria	Proteobacteria
12	1.8%	0.336	0.148	Klebsiella	Proteobacteria
13	1.8%	0.345	0.136	Citrobacter	Proteobacteria
14	1.3%	0.196	0.407	Actinobacillus	Proteobacteria
15	0.9%	0.355	0.125	Granulicatella	Firmicutes
16	0.9%	0.381	0.097	Enterobacter	Proteobacteria
17	0.8%	−0.158	0.507	Bacteroides	Bacteroidetes
18	0.8%	−0.483	0.031	Lachnoclostridium	Firmicutes
19	0.7%	0.319	0.170	Porphyromonas	Bacteroidetes
20	0.6%	0.270	0.250	Megasphaera	Firmicutes
21	0.6%	0.337	0.146	Lactobacillus	Firmicutes
22	0.6%	−0.460	0.041	Campylobacter	Proteobacteria
23	0.6%	0.338	0.145	Oribacterium	Firmicutes
24	0.5%	−0.237	0.316	Parascardovia	Actinobacteria
25	0.5%	−0.496	0.026	Ralstonia	Proteobacteria
26	0.3%	−0.003	0.991	Corynebacterium	Actinobacteria
27+	12.4%			all other genera	

Percentages represent average metagenomic abundance of the genus over all 20 subjects, as a percentage of all bacteria (col 2). Linear Pearson correlation of genus abundance with log of the DNA-Based total microbe count (col 3). The Pearson correlation coefficient was used to determine the *P*-value (column 4) for the null hypothesis that there is no correlation between genus abundance and bacterial load. Only Rothia, Lachnoclostridium, Campylobacter and Ralstonia differed significantly from the null hypothesis (shaded cells). Columns 5–8 display the genus and its taxonomy

### Species-level profiles

An average of 78% of DNA reads from single molecules were assigned to a species by the Illumina software [21]. In most cases, the assignment was confirmed when raw 16S rRNA V3-V4 interval gene sequences of a given species classification were retrieved and examined individually by multiple NCBI BLAST searches. There was generally over 98% sequence identity to a 16S rRNA exemplar for the species. This usually increased to 100% identity after the assembly of 10 or more sequence reads. Although the clustering of sequences was found to represent consistent OTUs, there were cases of systemic mis-identification of the OTU by the Illumina taxonomic system. One example was *Escherichia albertii,* which appeared as an abundant species in several subjects. Although the consensus sequence was a perfect match to *E. albertii*, the 16S rRNA V3-V4 interval was also identical to *E. coli* and species of the genus *Shigella*. PCR and QPCR with *E. coli*-specific primers of the *gyrA* gene were used to verify identity of this abundant species as *E. coli.* Another example was *Streptococcus tigurinus,* a newly discovered opportunistic pathogen [27] identified by the Illumina software. To confirm identity of this species, we retrieved 16 randomly chosen sets of *S. tigurinus* paired-end sequence reads from our raw sequence data and assembled these to generate a consensus. The consensus revealed a 100% match to Genbank exemplars of *S. tigurinus*, but also gave a 100% match to sequences of other viridans group *Streptococci* [28], including *S. mitis* and *S. oralis*. Given the consistently high abundance of this operational taxonomic unit (OTU) in the jejunum and in our saliva control, the widespread commensal *Streptococcus mitis* reported previously in the intestine [14] appears to be a more likely provisional species identity within the viridans group *Streptococci* [28]. Because of these two cases, taxonomy of each of the 45 most abundant species assigned by the Illumina software was checked by BLAST search of primary sequence data. This revealed mis-identification of a major fraction of *Haemophilus parainfluenzae* 16S rRNA gene sequence reads as *Mannheimia caviae*. Such errors may be intrinsic to the Illumina MiSeq metagenomics software or to its modified Greengenes 16S rRNA gene taxonomic database [21]. All primary, uncorrected Illumina abundance data used for the analysis is contained in 21 large excel S1-format files. These files are provided as “Additional files 1, 2, 3, 4, 5, 6, 7, 8, 9, 10, 11, 12, 13, 14, 15, 16, 17, 18, 19, 20 and 21”, and named according to alphabetical subject designations. Species identities confirmed, as well as those requiring taxonomic corrections, are also listed in the Additional files 22, 23 and 24.

To simplify the analysis of over 800 species identified per specimen, we selected the top 10 most abundant bacterial species of each patient. The abundance of a species within the “Top 10” typically ranged from 2% to 20% of all bacteria in that sample. We then asked how often a bacterial species ranked in the Top 10 among all 20 research subjects. Results are summarized in Table 4, which lists all 39 species found in the Top 10 set of at least one subject. A core set of ten species recurred in the Top 10 listings of at least 8 individuals, while 20 different species recurred in the Top 10 listings of at least 4 individuals. Half of the species in Table 4 reached Top 10 abundance in only one or two patients, suggesting considerable variation in jejunal microbiota at the individual species level. Many of these rarer species were sporadically very abundant, yet effectively absent in most other patients. It is of note that none of the subjects had significant numbers of virulent pathogen species often involved in acute gastroenteritis. *Staphylococcus aureus* was only detected in 3 patients at 0.05% or less. Common enteric pathogens such as *Clostridium difficile, Campylobacter jejunum,* and *Salmonella enterica* were not detected at all. *Pseudomonas* species were present, but in very low abundance, at 0.02% to 0.001% of total bacteria.

**Table 4:** Most abundant sequenced and cultured bacterial species

	Bacterial Species	Phylum	DNA: 16S analysis	Colonies
			Top 10 abundance	species detected
			% of Subjects	% of Subjects
1	*Streptococcus mitis (tigurinus,oralis)*	Firmicutes	100%	0
2	*Veillonella atypica*	Firmicutes	70%	0
3	*Haemophilus parainfluenzae*	Proteobacteria	65%	0
4	*Fusobacterium periodonticum*	Fusobacteria	60%	0
5	*Streptococcus vestibularis*	Firmicutes	55%	0
6	*Prevotella melaninogenica*	Bacteroidetes	50%	0
7	*Escherichia coli*	Proteobacteria	45%	25%
8	*Prevotella histicola*	Bacteroidetes	40%	0
9	*Streptococcus parasanguinis*	Firmicutes	40%	33%
10	*Streptococcus pseudopneumoniae*	Firmicutes	35%	0
11	*Actinomyces odontolyticus*	Actinobacteria	30%	8%
12	*Prevotella veroralis*	Bacteroidetes	30%	0
13	*Neisseria mucosa*	Proteobacteria	25%	0
14	*Veillonella dispar*	Firmicutes	25%	8%
15	*Prevotella pallens*	Bacteroidetes	25%	0
16	*Granulicatella adiacens*	Firmicutes	20%	0
17	*Rothia mucilaginosa*	Actinobacteria	20%	8%
18	*Actinobacillus parahaemolyticus*	Proteobacteria	15%	0
19	*Prevotella salivae*	Bacteroidetes	15%	0
20	*Gemella cunicula*	Firmicutes	10%	0
21	*Leptotrichia wadei*	Fusobacteria	10%	0
22	*Lachnoclostridium celerecrescens*	Firmicutes	10%	0
23	*Ralstonia pickettii*	Proteobacteria	10%	0
24	*Enterobacter soli*	Proteobacteria	10%	0
25	*Citrobacter freundii*	Proteobacteria	10%	17%
26	*Citrobacter werkmanii*	Proteobacteria	5%	0
27	*Campylobacter concisus*	Proteobacteria	5%	0
28	*Fusobacterium nucleatum*	Fusobacteria	5%	0
29	*Porphyromonas endodontalis*	Bacteroidetes	5%	0
30	*Corynebacterium argentoratense*	Actinobacteria	5%	0
31	*Klebsiella variicola*	Proteobacteria	5%	0
32	*Klebsiella granulomatis*	Proteobacteria	5%	0
33	*Megasphaera micronuciformis*	Firmicutes	5%	0
34	*Oribacterium sinus*	Firmicutes	5%	0
35	*Gemella sanguinis*	Firmicutes	5%	0
36	*Lactobacillus mucosae*	Firmicutes	5%	0
37	*Streptococcus salivarius*	Firmicutes	5%	58%
38	*Streptococcus lactarius*	Firmicutes	5%	0
39	*Streptococcus peroris*	Firmicutes	5%	0

DNA 16S analysis Top10: Fraction of subjects who ranked this species among their Top 10 most abundant, as determined by 16S rRNA metagenomic analysis of total bacterial DNA. Colonies: species detected. Fraction of subjects positive for the species by a spot-check sampling of colonies from rich medium aerobic and anaerobic cultures of the first 12 subjects recruited: A, C, D, G, I, L, N, O, P, R, T

The profile of bacterial species identified by metagenomics differed from that of colonies recovered in culture. Spot-check 16S rRNA identification of 45 aerobic and anaerobic bacterial colonies (Table 4, last column) indicated that *Streptococcus salivarius* was very common in live cultures, but was much less prominent in the 16S metagenomic analysis. In contrast, *Streptococcus mitis,* which was found in the Top 10 of all 20 subjects, did not appear at all among the 45 culture samples. A few species, such as *Escherichia coli and Streptococcus parasanguinis,* were abundant in both the metagenomic analysis and in the sampled colonies. This small-scale spot check suggested that there exist differences in the microbial viability or plating efficiency between species, even within the same genus.

### Variation in species abundance

Table 5 presents relative abundance of 9 selected abundant species in all 20 jejunal samples and in the oral positive controls. In contrast to the consistent Top 10 abundance of *Streptococcus mitis,* other species showed a more sporadic pattern, with a high fraction in some patients, and near absence in others. Pathogenic bacterial infections are often characterized by a large and broad-based decrease in species diversity, in part due to the dominance of a small number of species [8, 9, 28, 29]. To determine whether the sporadic high abundance of single species was associated with similar decreases in species diversity, each sample was analyzed for intrinsic alpha species diversity by means of the Shannon index and inverse of the Simpson index (Inverse-Simpson). These were calculated for the top 200 species in the raw Illumina species classification. All jejunal samples showed lower diversity than the two oral samples, but none showed large changes in the Shannon index (Table 5). However, we observed substantial differences in the more sensitive Inverse-Simpson index, but these were not generally associated with over-representation of a single species. The one exception was *E. coli*, which showed a substantial negative Pearson correlation coefficient of *r* = − 0.644 and a *p*-value of 0.0022. This indicated that an increased fraction of *E. coli* in the population was likely associated with decreased microbial diversity. In contrast, the abundant commensal *Streptococcus mitis* revealed no significant correlation, with *r* = − 0.103 and a *p*-value of 0.67. To test whether subjects with higher levels of total jejunal bacteria exhibited a reduced diversity, we measured the correlation of species diversity with the log of bacterial load. In this case, we found no significant correlation, with Pearson *r* = 0.254 and a *p*-value of 0.280 for the null hypothesis. By the criterion of decreased species diversity, we found no evidence that higher bacterial load is associated with dysbiosis in the jejunum.

**Table 5:** Species composition and diversity

			Gram-positive bacteria			Gram-negative bacteria					
			Firmicutes			Proteobacteria				Fusobacteria	Bacteroidetes
	Microbial Diversity		*Strep.*	*Strep.*	*Strep.*	*Haemoph.*	*Escherichia*	*Citrobacter*	*Klebsiella*	*Fusobact.*	*Prevotella*
Subject	Shannon	Inv-Simpson	*mitis*	*vestibularis*	*pseudopn.*	*parainflu.*	*coli*	*freu. + werk.*	*varii. + gran.*	*periodont.*	*melaninogen.*
A	3.57	19.49	2.31	1.22	0.49	0.17	0.01	0	0	8.26	6.19
B	3.23	13.20	2.16	0	0.23	0.33	0.03	8.61	31.60	1.59	1.12
C	3.36	14.09	9.62	0	1.63	1.63	9.67	0	0.01	2.37	0.67
D	3.25	9.05	2.07	0	0.32	16.52	1.50	1.22	0.49	5.10	0.13
E	3.44	16.91	2.64	9.60	1.13	0.26	0.25	20.48	0.15	0.08	6.19
F	3.11	14.42	8.75	3.02	3.43	0.05	0	0	0	0	0.08
G	3.73	16.62	11.51	0	1.50	3.60	3.52	0	0.01	0.69	1.30
H	3.26	12.89	11.53	1.03	1.70	4.97	0.62	0.01	0.01	13.30	2.22
I	3.33	12.26	14.06	0	2.05	0.08	2.19	0	0	0.59	0.14
J	3.55	16.81	3.33	4.29	1.07	3.33	0	0	0	0.65	12.81
K	2.59	4.31	3.09	1.35	0.92	0.84	34.47	0	0.01	4.49	0.76
L	3.77	22.00	7.49	1.36	3.34	1.93	0.62	0	0.02	0.33	8.16
M	3.55	17.85	11.94	7.58	2.04	0.00	3.00	0.01	0.02	0.13	4.07
N	2.94	8.19	12.20	0.11	7.43	0.57	0.04	0.01	0	7.08	21.25
O	3.40	12.62	3.80	1.34	1.09	1.00	0	0	0	1.92	16.16
P	3.37	16.16	2.26	5.00	1.09	1.43	0	0	0	0.61	11.10
Q	3.75	6.05	13.98	0	2.25	0.63	7.23	0	0.01	1.93	1.28
R	2.95	6.68	2.70	0	0.38	2.94	27.04	0	0.02	0.44	0.10
S	3.12	10.21	4.84	0	0.78	0.04	13.96	0	0.01	0.17	0
T	3.54	19.38	2.68	1.58	0.90	2.37	0	0	0	2.92	8.80
Plaque	3.82	24.05	0.61	0.01	0.07	0.20	0	0	0	0.07	0.31
Saliva	3.83	23.56	2.16	0.14	0.21	2.72	0.02	0.01	0.03	0.54	1.38

Subjects are listed from highest to lowest bacterial load. Microbial species diversity: The Shannon index and the inverse of the Simpson index were calculated for the 200 most abundant OTUs. Selected Bacterial Species: The percentage of total bacteria represented by the species. Columns: 4. *Streptococcus mitis*; 5. *Streptococcus vestibularis*; 6. *Streptococcus pseudopneumoniae*; 7. *Haemophilus influenzae*; 8. *Escherichia coli*; 9. *Citrobacter freundii* + *C. werkmannii*; 10. *Klebsiella variicola* + *K. granulomatis*; 11. *Fusobacterium periodonticum*; 12. *Prevotella melaninogenica*

## Discussion

### Similarity to oral microbes and major differences from the colon

Of the 20 subjects, 19 had a jejunal phylum profile that was very similar to those of the mouth [26] and stomach [23], both by inspection and by their clustering in a principal components analysis plot. In contrast, these jejunal samples had phylum profiles that differed considerably from the colon [24, 25]. Bacteria of the phyla Proteobacteria, Actinobacteria and Fusobacteria are typically rare in the adult colon but abundant in both our oral controls and in 19 of the jejunal samples. Although the feces of infants are rich in Proteobacteria, including *Escherichia coli* [8], the phylum Proteobacteria is present but relatively rare in the microbiome of the adult colon [13]. The only jejunal outlier in the PCA analysis, subject F, had a Firmicutes-abundant phylum profile superficially typical of colonic bacteria, but further examination at the genus and species level revealed that it lacked extreme obligate anaerobes belonging to the class Clostridia, which are characteristic of the colonic bacteria. Instead, the Firmicutes component of subject F was more typical of oral bacteria, with an abundance of the class Bacilli, which includes facultative anaerobes such as the Streptococci.

### Genus and species level analysis reveal differences from oral microbiota

**Fig. 6:**
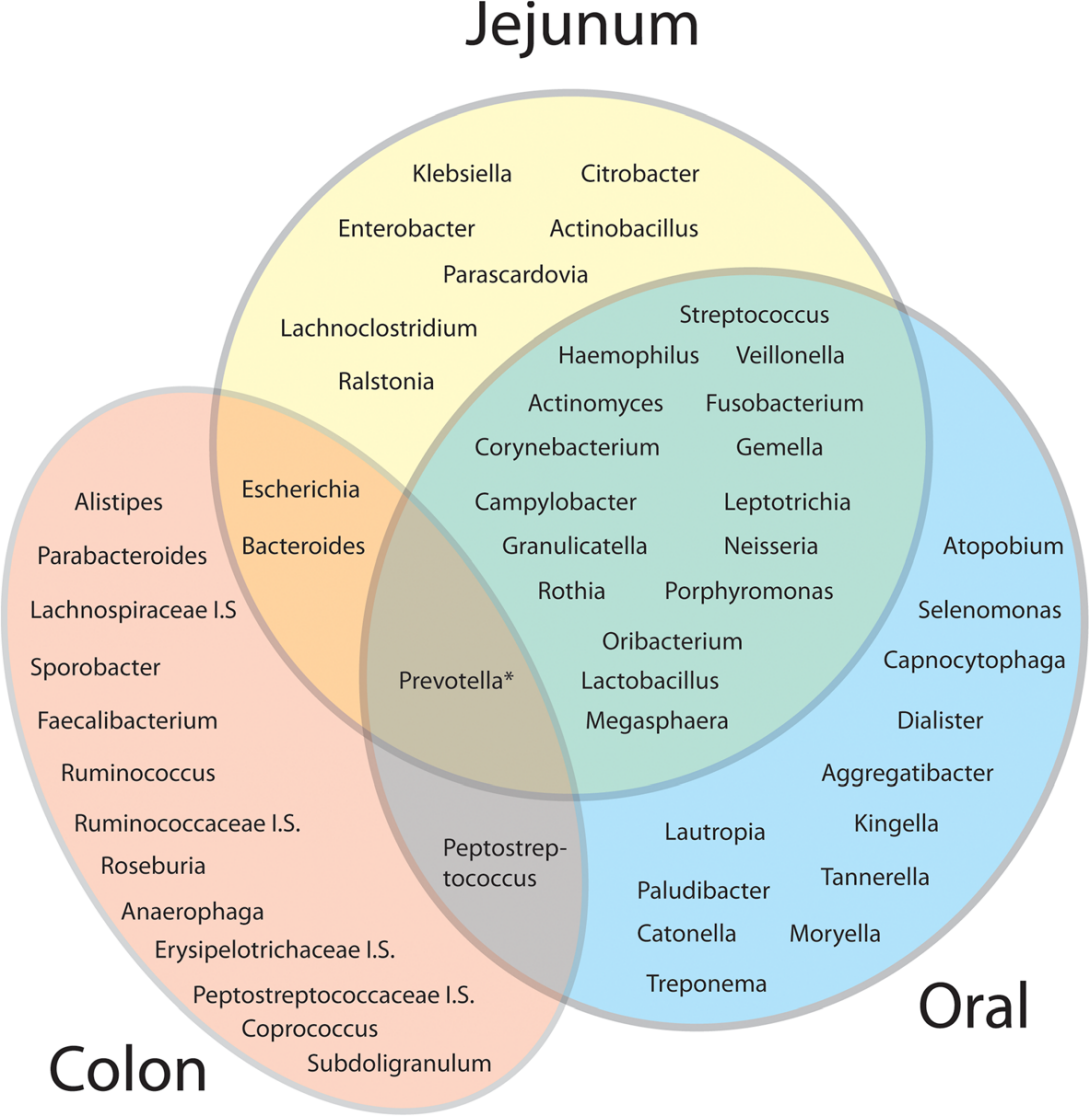
Major bacterial genera of the jejunum in relation to oral and colonic microbiota. The 26 most abundant species in our 20 patients are shown in the central circle of the Venn diagram. Genera in and overlapping the red ellipse are derived from published metagenomic studies of colonic bacteria [24, 25]. The blue ellipse represents the most abundant genera of oral microbes, obtained from our saliva and plaque control samples, and from references [22, 26] and [30]

Although the phylum profile showed great similarity with oral bacteria, analysis at the species and genus level indicated substantial differences. A recurring core of abundant species in the jejunum belonged to the genera *Streptococcus, Veillonella, Prevotella, Fusobacterium, Escherichia* and *Haemophilus* (Tables 4, 5). The 26 most abundant genera (Table 3) are shown in the central yellow circle of the Venn diagram of Fig. 6. Of these, 17 are shared with the 30 most abundant oral genera. The blue ellipse summarizes our oral control data, and previously published data [26, 30]. Of 17 major genera of the adult colon [24, 25] shown in the red ellipse, only 3 overlap with the jejunum. Only *Prevotella*, a genus containing oxygen-tolerant obligate anaerobes, was abundant in all three of these bacterial populations. Genera of the Enterobacteriaceae, such as *Escherichia, Citrobacter* and *Klebsiella* are rarely found in the adult oral microbiota, but were often abundant in the jejunal lumen. The 12 genera exclusive to the mouth and absent or rare within the jejunum and colon include species that were enriched in our samples of dental plaque relative to saliva. These grow vigorously as biofilms anchored to the solid surfaces of the teeth, and are continuously shed into the saliva (Fig. 1). The deep sub-gingival crypts surrounding the teeth provide additional anaerobic microenvironments that are not present in the jejunum, which contains only a soft mucosal epithelium. The jejunal samples varied in Shannon and Simpson species diversity. However all jejunal samples were lower in Shannon and Simpson diversity than our saliva and dental plaque samples (Table 4).

### Do the jejunal lumen and mucosa contain the same population of bacteria?

Although the phylum profile for bacteria in the jejunal lumen varied between individuals, we found that its average value (Fig. 3b: Av) was surprisingly similar to the average value obtained in an earlier metagenomic study of bacteria in the jejunal mucosa (reference [15], Fig. 3b: R1). This similarity extended to genus-level composition (Table 3). The earlier study focused on mucosa [15] because of the expectation that the mucosal bacteria would be different from those of the lumen. There is a precedent for such differences in the colon, where it is well established that mucosa-bound bacteria have a different species mixture from the lumen [31, 32]. In the colon, the mucosal layers are partially oxygenated due to their proximity to the blood capillaries of the gut epithelium. In contrast, the dense facultative anaerobes of the colonic lumen consume the available oxygen and create an environment much more favorable to the growth of extreme anaerobes [32]. Our results raise the question of whether there could be greater similarities between microbiota of the jejunal mucosa and lumen. In the future, this could be addressed by simultaneously collecting microbes from both sources. Because of its much lower density of bacteria, the lumen is likely to remain oxygenated at similar levels to the adjoining jejunal mucosa. Given our observations of the low density and variable viability of bacteria in the jejunal lumen, it is also possible that jejunal bacteria grow endogenously in the mucosal layer, and are shed into the lumen. They would persist there, until transported by peristalsis to lower levels of the alimentary tract.

### Basis for variation in bacterial viability

Our observation of large differences in apparent viability of jejunal bacteria (Table 1, Fig. 1a) did not correlate with differences in the abundance of species or genera. This is in contrast to the colon, where 16S sequencing identified many new species that grew poorly or not at all in standard microbial culture [5, 13]. In the jejunum, lower plating efficiency of certain species or genera did not explain the large differences in viability. For example, some jejunal samples exhibiting very low viability were abundant in DNA from *E. coli*, a species that is easily cultured. For these samples, it seems more plausible that a hostile environment, possibly due to an unfavorable combination of bile salt, pancreatic enzymes, low pH, or other factors, could have decreased bacterial viability in the lumen. It remains an open question whether non-viable 16S QPCR-detected DNA has been released by bacterial lysis, or is confined to intact but dead or inactivated cells. Due to the general abundance of pancreatic DNAase and other digestive nucleases in the jejunum, DNA released from broken bacteria should have been rapidly degraded.

### High bacterial load is not associated with increases in colon-specific bacteria

Bacterial genus composition of the jejunum shows very little overlap with that of the colon. One exception is *Prevotella*, which includes obligate anaerobes that are also oxygen tolerant, and can grow in oral, jejunal or colonic environments. However, most colonic microbes are not suited to growth in the partially oxygenated environment of the jejunum. *Faecalibacterium prausnitzii* normally constitutes a total of 4% to 8% of total bacterial DNA [31]. This species and other extreme obligate anaerobes were essentially absent (< 0.0005%) from all our DNA samples from the jejunum. Only a small amount of retrograde flow would be necessary to cause considerable increases in DNA of these microbes in the relatively sparse population of the jejunum. DNA of colonic bacteria should persist long enough to be detected, even if they were non-viable, and there were no growth of these migrants within the jejunum. Jejunal samples showing considerable bacterial excess, postulated to result from retrograde migration of gut bacteria, showed no evidence of DNA from these colonic extreme anaerobes, either at the phylum, genus or species level. These results strongly suggested that, among our 20 subjects, there was little, if any, retrograde mass transport of colonic contents into the jejunum. This is of interest because there are many conditions that affect gut motility, and could, in principle, allow such retrograde movement [4]. It should be noted that our study excluded subjects who had lost their iliocaecal valve due to surgery. For such individuals, the valve defect could allow some retrograde flow of bacteria from the colon, producing elevated bacteria in the jejunum. In the future, it might be possible to monitor the magnitude of this retrograde flow after surgery by detecting the presence of *Faecalibacterium prausnitzii* and other colonic species in the jejunum.

### Does a higher bacterial load in the small intestine indicate bacterial infection or dysbiosis?

The complete absence of virulently pathogenic microbes such as *Salmonella typhimurium*, *Campylobacter jejuni* and *Clostridium difficile* supported the clinical determination that none of the subjects had acute gastroenteritis. The opportunistic pathogen *S. aureus* was detected in the jejunum, but only at very low levels in a few subjects. However, we did observe recurrent major overgrowths of other opportunistic pathogens including *Eschericha coli*, *Klebsiella variicola* [33]*,* and *Citrobacter freundii* [34]. An indicator that often accompanies infections or bacterial blooms is a sharp decrease in the diversity of microbial species, which can be measured by calculating the Shannon index or the more sensitive Inverse-Simpson index [29, 35]. One prominent example of a chronic microbial infection is colonization of the stomach by *Helicobacter pylori*, a species associated with ulcers and gastric cancer [23]. In a recent metagenomic study of bacteria in the stomach [35], most individuals without *H. pylori* had an average Shannon diversity of 3.01, while those with a chronic *H. pylori* infection had a greatly reduced Shannon diversity index of 0.305. In a recent study of *Clostridium difficile* infection of the gut, the fecal microbiome of normal controls had an average Shannon index of 3.16, while patients with recurrent *C. difficile* infections had a reduced Shannon index of 1.56 [29].

In our study of the jejunum, none of the subjects exhibited such major decreases in the Shannon index (Table 5). Our saliva and dental controls represented a high diversity of bacterial species. These had a Shannon index of 3.82 and 3.83, while diversity of the 20 jejunal samples ranged from a high of 3.77 to a low of 2.59. Even with the more sensitive Inverse-Simpson diversity index, increases in total bacterial load were not significantly associated with decreases in species diversity. The one exception was *Escherichia coli.* Increased representation of *E. coli* in the jejunum showed a strong and highly significant (*P* = 0.0022) correlation with a decrease in Inverse-Simpson species diversity. This suggested that abundance of *E. coli* in the adult jejunum could be associated with mild dysbiosis. Whether it is associated with any clinical symptoms remains to be determined.

## Conclusions

Our study has successfully used standard clinical enteroscopy to sample and characterize the normally sparse bacterial community of the jejunal lumen. 16S rRNA gene metagenomic analysis has revealed a jejunal microbiome with a common core of bacterial species, characterized by an abundance of *Streptococcus* along with other facultative anaerobes and oxygen-tolerant obligate anaerobes. Although the jejunal bacterial microbiota showed similarities to those of the oral cavity, this population was distinguished by the presence of a varying assortment of Enterobacteriaceae, including *Eschericha, Citrobacter and Klebsiella*. However, the species profile of microbial DNA provided no evidence of significant retrograde transfer of bacteria from the colon to the jejunum, even in those subjects with much higher bacterial loads. Although total bacterial DNA load ranged over 100-fold and CFUs ranged over 1000-fold, higher bacterial loads were not associated with decreased Shannon species diversity. This suggested that a high level of jejunal bacteria in reasonably healthy subjects was not associated with classic dysbiosis or infection. We hope that this work will provide a basis for future studies involving chronic dysfunction and acute infection of the jejunum.

## Additional files

Additional file 1: Oral-saliva. Illumina abundance summary file. Distribution of microbiota from raw Illumina file. (CSV 101 kb)

Additional file 2: A. Illumina abundance summary file. Distribution of microbiota from raw Illumina file. (CSV 103 kb)

Additional file 3: B. Illumina abundance summary file. Distribution of microbiota from raw Illumina file. (CSV 98 kb)

Additional file 4: C. Illumina abundance summary file. Distribution of microbiota from raw Illumina file. (CSV 101 kb)

Additional file 5: D. Illumina abundance summary file. Distribution of microbiota from raw Illumina file. (CSV 127 kb)

Additional file 6: E. Illumina abundance summary file. Distribution of microbiota from raw Illumina file. (CSV 103 kb)

Additional file 7: F. Illumina abundance summary file. Distribution of microbiota from raw Illumina file. (CSV 101 kb)

Additional file 8: G. Illumina abundance summary file. Distribution of microbiota from raw Illumina file. (CSV 133 kb)

Additional file 9: H. Illumina abundance summary file. Distribution of microbiota from raw Illumina file. (CSV 106 kb)

Additional file 10: I. Illumina abundance summary file. Distribution of microbiota from raw Illumina file. (CSV 113 kb)

Additional file 11: J. Illumina abundance summary file. Distribution of microbiota from raw Illumina file. (CSV 88 kb)

Additional file 12: K. Illumina abundance summary file. Distribution of microbiota from raw Illumina file. (CSV 98 kb)

Additional file 13: L. Illumina abundance summary file. Distribution of microbiota from raw Illumina file. (CSV 105 kb)

Additional file 14: M. Illumina abundance summary file. Distribution of microbiota from raw Illumina file. (CSV 111 kb)

Additional file 15: N. Illumina abundance summary file. Distribution of microbiota from raw Illumina file. (CSV 109 kb)

Additional file 16: O. Illumina abundance summary file. Distribution of microbiota from raw Illumina file. (CSV 95 kb)

Additional file 17: P. Illumina abundance summary file. Distribution of microbiota from raw Illumina file. (CSV 109 kb)

Additional file 18: Q. Illumina abundance summary file. Distribution of microbiota from raw Illumina file. (CSV 126 kb)

Additional file 19: R. Illumina abundance summary file. Distribution of microbiota from raw Illumina file. (CSV 128 kb)

Additional file 20: S. Illumina abundance summary file. Distribution of microbiota from raw Illumina file. (CSV 115 kb)

Additional file 21: T. Illumina abundance summary file. Distribution of microbiota from raw Illumina file. (CSV 112 kb)

Additional file 22: Relative Abundance of 26 Major Genera in the Jejunum of Individual Subjects by 16S Metagenomic Analysis. Genus level distribution for each of the 20 subjects. (XLSX 16 kb)

Additional file 23: Corrections required for identifications by Illumina MiSeq Reporter v2.5 software package. This is a list of corrections of species reassignments that need to be applied to correct the Illumina Reporter v2.5 classification and abundance data in the attached Tables A through T. (DOC 25 kb)

Additional file 24: Illumina raw abundance data for operational taxonomic units. Explains the corrections file (Additional file 23) and Illumina abundance summary data files A through T. (DOCX 13 kb)

## Abbreviations

16S rRNA: 16 Svedberg unit sedimenting ribosomal RNA
BGE: Bacterial genome equivalent
CDC-Blood: Center for Disease Control blood agar
CFU: Colony forming units
KV-Blood: Kanamycin-vancomycin blood agar
Levine-EMB: Levine eosin-methylene blue agar
NCBI-BLAST: National Center for Biotechnology-Basic Local Alignment Search Tool
OTU: Operational taxonomic unit, a provisional classification of DNA sequence
PCR: Polymerase chain reaction
PEA-Blood: Phenylethyl alcohol blood agar
QPCR: Quantitative PCR
SIBO: Small intestinal bacterial overgrowth
