# Supplementary material for: The human jejunum has an endogenous microbiota that differs from those in the oral cavity and colon
Source: BMC Microbiol. 2017 Jul 17;17:160. doi: 10.1186/s12866-017-1059-6 (PMC5513040; doi:10.1186/s12866-017-1059-6)
Supplement: Supplementary file 23 — Corrections required for identifications by Illumina MiSeq Reporter v2.5 software package. This is a list of corrections of species reassignments that need to be applied to correct the Illumina Reporter v2.5 classification and abundance data in the attached Tables A through T. (DOC 25 kb) [file 12866_2017_1059_MOESM23_ESM.doc]

**Corrections required for identifications by Illumina MiSeq Reporter v2.5 software package**

ID in red - seriously compromised-need to be removed

ID in green - needs only re-assignment to new species or genus.

ID in blue - no changes required

Actinobacillus parahaemolyticus => OK no change

Actinomyces lingnae => Change to Actinomyces odontolyticus

Actinomyces odontolyticus => OK no change

Bacteroides denticanum => Remove Species

Butyrivibrio proteoclasticus => Change to Lachnoclostridum celerecrescens

Campylobacter concisus => OK no change

Chitinophaga soli => Change to Lachnoclostridum celerecrescens

Citrobacter freundii => OK no change

Citrobacter werkmannii => OK no change

Fusobacteriium naviforme => Change to Fusobacterium periodonticum

Fusobacterium nucleatum => OK no change

Fusobacterium Periodonticum => OK no change receive additional counts from F. naviforme

Gemella cuniculi => OK no change

Granulicatella adiacens => OK no change

Escherichia albertii => Escherichia coli

Haemophilus pseudopneumoniae => OK no change receive additional counts from M. caviae

Klebsiella variicola => OK no change

Klebsiella granulomatis => OK no change

Leptotrichia wadei => OK no change

Mannheimia caviae => Change to Haemophilus pseudopneumoniae

Megasphaera micronuciformis => OK no change

Neisseria mucosa => OK no change

Oribacterium sinus => OK no change

Pediococcus => Remove Genus and Species

Phenylobacterium lituiforme => Remove only 78% to 77% identity for Phenylobacterium genus

Porphyromonas endodontalis => OK no change

Prevotella oris => Remove Species

Prevotella histicola => OK no change

Prevotella pallens => OK no change

Prevotella veroralis => OK no change

Ralstonia insidiosa => Change to Ralstonia pickettii

Rothia mucilaginosa => OK no change

Serratia entomophila => erroneous Remove Species and Genus

Streptococcus bovis => Change to S. parasanguinis

Streptococcus infantis => remove - indistinguishable from S. mitis

Streptococcus parasanguinis => OK no change receive additional counts from S. bovis

Streptococcus peroris => OK no change

Streptococcus pseudopneumoniae => OK no change

Streptococcus tigurinus => change to S. mitis (same as S.oralis, S.mitis)

Streptococcus vestibularis => OK no change

Tolumonas auensis => Remove - only 90% match to reference genome T. auensis

Veillonella atypica => OK no change

Veillonella dispar => OK no change (include V. parvula)

Veillonella parvula => Veilllonella dispar
