# Supplementary material for: The human jejunum has an endogenous microbiota that differs from those in the oral cavity and colon
Source: BMC Microbiol. 2017 Jul 17;17:160. doi: 10.1186/s12866-017-1059-6 (PMC5513040; doi:10.1186/s12866-017-1059-6)
Supplement: Supplementary file 24 — Illumina raw abundance data for operational taxonomic units. Explains the corrections file (Additional file 23) and Illumina abundance summary data files A through T. (DOCX 13 kb) [file 12866_2017_1059_MOESM24_ESM.docx]

**Illumina raw abundance data for operational taxonomic units.**

All primary, uncorrected Illumina abundance data on which the metagenomic analysis of the paper is based is contained in 21 large excel S1-format files. These are named according to patient designations in order of increasing jejunal CFUs (20 files a through t) and for the oral saliva positive control. Number of hits represents the number of cluster sequences that could be classified. Percent indicates the portion of total hits an estimate of raw abundance (including the unclassifieds as part of the total percentage). Unclassified sequences are generally found to be non-bacterial human genome sequences.

While the most abundant species are correctly assigned, some of the initial taxonomic assignments are incorrect in these raw files. These have been corrected using the raw sequence data, as discussed in the paper. The "ID corrections" file provides a guide to the corrections required after manual BLAST curation.
